# Redo Aortic Surgery for Angulated Grafts: How Far Should We Go?

**DOI:** 10.1016/j.jaccas.2025.106729

**Published:** 2026-06-10

**Authors:** Niti Dalal, Aabha Divya

**Affiliations:** a Department of Epidemiology, Tulane School of Public Health and Tropical Medicine, New Orleans, Louisiana, USA; b Division of Cardiothoracic Surgery, Tulane University School of Medicine, New Orleans, Louisiana, USA

**Keywords:** anticoagulation, aorta, cardiac magnetic resonance, dissection, stroke

**Figure:**
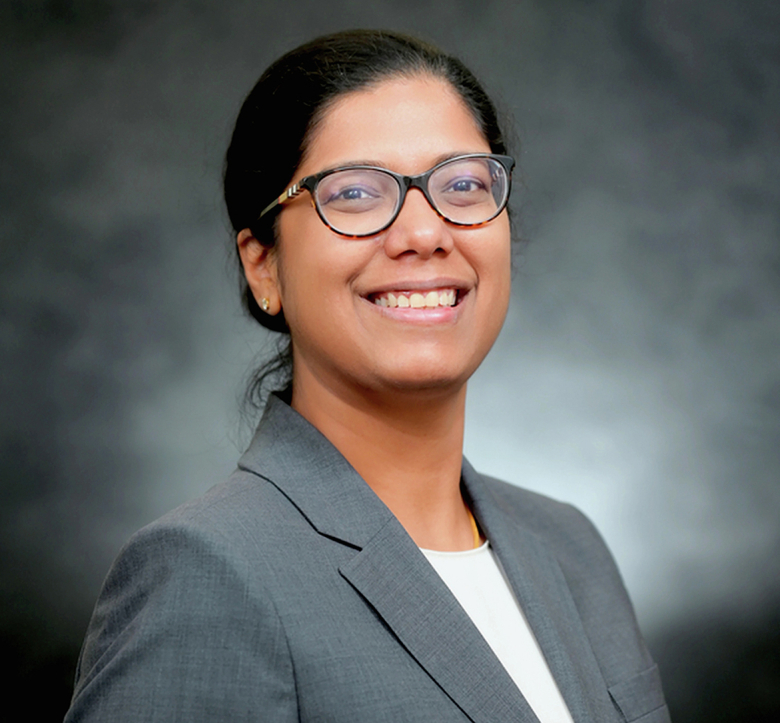

**Visual Summary:**
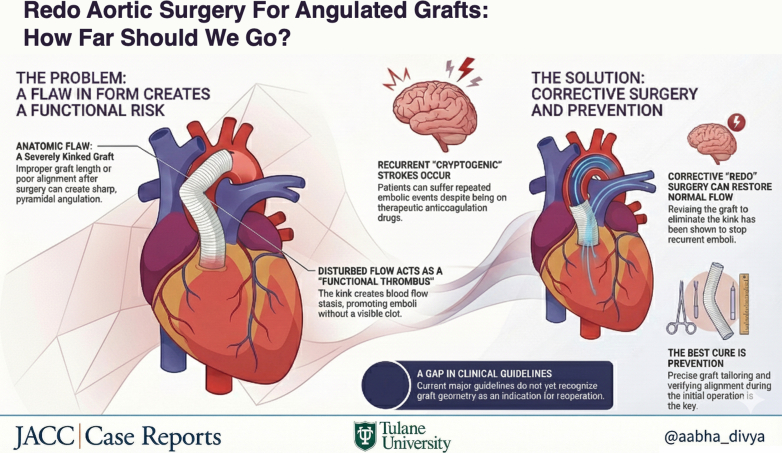
Severe Angulation of an Ascending Aortic Graft Can Create Disturbed Flow and Functional Stasis, Leading to Recurrent Embolic Events Despite Therapeutic Anticoagulation and the Absence of Visible Thrombus In selected patients, a redo surgery correcting graft geometry may restore physiologic flow and prevent further embolic complications.

Among the many lessons of aortic surgery, one of the most enduring is that form dictates function. Severe pyramidal angulation or kinking of the ascending graft can distort flow patterns and predispose to thromboembolism even when no clot is visualized.

In this issue, Timmermans et al. describe a patient who suffered recurrent ischemic strokes over 4 years after Type A dissection repair, despite being on therapeutic anticoagulation. Electrocardiography-gated computed tomography angiography revealed a severely kinked ascending graft without thrombus. Redo surgery correcting graft geometry eliminated recurrent embolic events during follow-up.^1^ This report highlights that disturbed flow can be as dangerous as a clot, challenging the long-standing assumption that visible thrombus is required to justify high-risk reoperation.

Normal aortic flow is laminar and helical, which helps maintain uniform wall shear stress and protects against thrombus formation.^2^^,^^3^

• Computational fluid dynamics and 4D flow magnetic resonance imaging can visualize disturbed flow and quantify kinetic energy loss, offering mechanistic insight when traditional imaging is inconclusive.
• Computational fluid dynamics and 4D flow magnetic resonance imaging studies demonstrate that sharply angulated prosthetic segments produce low-velocity recirculation zones and oscillatory shear, which, in turn, promote platelet activation and fibrin deposition.

In this case, the severely kinked graft created a segment of relative stasis, which resulted in a “virtual thrombus.” Such disturbed hemodynamics likely sustained a proembolic environment despite therapeutic rivaroxaban levels.

Redo ascending aortic surgery is inherently a high-risk procedure, and surgeons would traditionally wait for objective evidence, such as thrombus, pseudoaneurysm, or anastomotic failure, before reintervention. However, in the current case, no thrombus was identified on imaging or intraoperatively, and yet recurrent embolic events persisted under optimal anticoagulation. After graft revision and elimination of the kink, the patient remained stroke-free. There have been similar reports in the literature:

• Hasami et al^4^ described stroke secondary to a severely kinked graft with mural thrombus that resolved after surgical correction.
• Wahba et al^5^ reported late thrombosis in a kinked ascending graft despite adequate anticoagulation.
• Saitoh et al^6^ reported a case involving a severely angulated graft resulting in hemolysis and severe anemia.

All these cases outline a spectrum from disturbed flow to the presence of thrombus caused by geometric distortion.

However, acknowledging a conservative perspective, one could argue that a redo surgery should be reserved for patients with objectively documented pathology. In the absence of these findings, the morbidity and mortality associated with reoperation may outweigh the theoretical benefits of correcting graft geometry. Anticoagulation and close imaging follow-up remain sufficient in most cases, and as mentioned, the mechanistic link between kinking without thrombus and clinical events remains largely based on anecdotal reports. Current guidelines reflect this and do not address graft geometry^7^, ^8^, ^9^, ^10^:

• The 2022 American College of Cardiology/American Heart Association Guideline for the Diagnosis and Management of Aortic Disease emphasizes lifelong surveillance and individualized decision-making but offers no recommendations regarding graft geometry or angulation as an indication for reoperation.
• Similarly, the 2022 AHA Scientific Statement on Imaging and Surveillance of Chronic Aortic Dissection underscores the need for standardized follow-up and mentions novel hemodynamic imaging but does not address prosthetic configuration.
• The 2014 European Society of Cardiology Guidelines on the Diagnosis and Treatment of Aortic Diseases provide comprehensive recommendations for imaging, surgical thresholds, and long-term surveillance; however, they do not include a reference to graft configuration or angulation as an indication for reoperation.
• The European Association for Cardio-Thoracic surgery (EACTS) Expert Consensus on the Management of Type A Aortic Dissection outlines detailed surgical and follow-up strategies; however, reoperation criteria are limited to aneurysmal dilation, pseudoaneurysm, or prosthetic infection.

Despite this, the case by Timmermans et al and corroborating reports suggest that a subset of patients may benefit from a geometry-aware perspective. Recurrent embolic events despite optimal anticoagulation indicate that disturbed flow can act as a functional thrombus. In addition, no consensus exists for antithrombotic management when embolic events recur without thrombus. Most centers default to single antiplatelet therapy after ascending repair unless another indication exists.

Ultimately, the best management is prevention. Excessive graft length, misalignment at the sinotubular junction, and neglect of the natural curvature predispose to postoperative “pyramidal” configuration. Key preventive principles include precise tailoring of graft length, preservation of the native curvature of the ascending aorta, the use of separate or beveled graft segments at sharp bends, and intraoperative confirmation after refilling the graft under physiologic pressure to reveal redundancy before closure. Attention to graft alignment during the index operation remains the most effective prophylaxis against late embolic complications.

This case reinforces that geometry itself can be independently pathological. Disturbed flow may act as a functional thrombus, justifying surgical correction even in the absence of a visible clot. While conservative management remains appropriate for many, adopting a geometry-aware framework can improve outcomes. As imaging and computational modeling advance, incorporating geometric and hemodynamic assessments into postoperative surveillance could prevent devastating “cryptogenic” strokes after aortic surgery.

## Funding Support and Author Disclosures

The authors have reported that they have no relationships relevant to the contents of this paper to disclose.
